# Knowledge, Attitude, and Perception of Oral Health Among Parents of Children at Risk of Infective Endocarditis: A Cross-Sectional Study

**DOI:** 10.7759/cureus.44855

**Published:** 2023-09-07

**Authors:** Faisal S AlSuliman, Saleh A Alajlan, Omer A Algonaid, Lama Y Almashham, Rahaf H Alawaji

**Affiliations:** 1 Orthodontics, King Fahad Medical City, Riyadh, SAU; 2 Pediatric Dentistry, King Fahad Medical City, Riyadh, SAU; 3 Pediatric Cardiology, King Fahad Medical City, Riyadh, SAU; 4 General Dentistry, King Saud University, Riyadh, SAU

**Keywords:** oral health, pediatric, dental caries, oral hygiene, infective endocarditis

## Abstract

Background: Infective endocarditis (IE) is a serious complication primarily affecting patients with cardiac conditions. It is widely recognized that oral microorganisms contribute to the development of IE.

Objectives: The objective is to assess the knowledge, attitudes, and perception of parents of children at risk of IE by studying the preventive measures employed by them.

Materials and methods: A self-constructed questionnaire composed of 14 questions was distributed among parents of children aged 0-12 years with known cardiac diseases. The parents/caregivers who were attending the pediatric outpatient clinics at King Fahad Medical City (KFMC) were selected randomly.

Results*: *A total of 112 parents responded to this questionnaire. Almost 50% of the participants in this study reported that their children do not brush their teeth regularly, with only 12.5% of them brushing twice a day. Regarding the role of parents in supervising oral hygiene, 62.5% of them stated that they only provide advice but do not watch their child brush their teeth, and only 9.8% of parents are actively involved in advising and watching their children brush their teeth. In this study, 95.5% of parents agreed that maintaining good dental health was crucial for overall body health, and 58% of the participants showed interest in receiving more education about oral health and its impact on overall well-being. To assess the statistical significance, a nonparametric Pearson's chi-square test for fitness was employed. A p-value of ≤ 0.05 was used to report the statistical significance of the results.

Conclusion*: *While the parents demonstrated adequate knowledge of oral health, their attitudes toward it were lacking. So, both parents and children require a modification in their attitudes toward dental care and oral health.

## Introduction

Infective endocarditis (IE) is a serious complication primarily affecting patients with cardiac conditions [1], such as those with congenital heart disease, valve replacements, cardiac implantable electronic devices, chronic rheumatic heart disease, nosocomial infection, and poor oral hygiene [2]. Although it is considered rare, it is even less common in children. It is estimated that only 0.43-0.69 cases occur per 100,000 children annually [3,4]. However, the mortality rate is still high, ranging from 1 to 5% in children and 10% in adults [3-5]. Due to the rise in risk factors, an increase in incidence has been seen globally in recent decades. In fact, in cases of bacteremia, younger age is a predisposing factor for IE, and younger patients are more likely to experience unfavorable outcomes [1,4,6]. It is well established that oral microorganisms play a role in the development of IE. Streptococci, particularly S. viridans, are the predominant causative microorganisms in most cases of patients with positive blood cultures [7]. Even under normal physiological conditions, inadequate oral hygiene can lead to bacteremia, increasing the risk of permanent disease acquisition [8]. Therefore, individuals at risk are strongly advised to establish and maintain meticulous oral health habits to minimize potential sources of bacteremia [9,10]. Limited literature exists regarding the oral health of children with pre-existing congenital heart disease, but studies have shown that these children experience poorer oral health compared to control groups [11-13]. Risk factors include frequent consumption of sugared medicines, increased susceptibility to developmental enamel defects [12], and negligence of oral hygiene due to prioritizing their cardiac condition over meticulous oral care [11-14].

However, the importance of assessing the oral health of children at risk for this condition is often overlooked, highlighting the growing need to evaluate the current knowledge, attitude, and perception among parents regarding this issue. Studies in Saudi Arabia about this issue are scarce; hence, the objective of this study is to obtain an up-to-date assessment of the knowledge and preventive measures employed by parents of children at risk of IE.

## Materials and methods

A cross-sectional study was conducted using a survey questionnaire that was distributed randomly to obtain responses from parents/caregivers of children with known cardiac diseases, as specified by the American Heart Association (AHA) conditions associated with the risk of IE (2017). The age of the children ranged from infancy to 12 years, attending the pediatric outpatient clinics at King Fahad Medical City (KFMC) over a period of seven weeks (October 10th to November 25th, 2022). Patients with other chronic medical conditions were excluded from this study. The objective of the study was to assess the knowledge and preventive measures employed by parents/caregivers using a validated self-constructed questionnaire.

Data collection

Data for the survey were collected using the convenience sampling technique. Parents were approached on the day of their child’s appointment at the waiting area of the clinic. Participation was voluntary, and informed consent was obtained beforehand. Personal information was not collected to ensure the anonymity of the participants. Patient’s electronic medical files were reviewed to verify the child’s cardiac conditions according to AHA criteria. The parents/caregivers received a full explanation of how to answer the questionnaire. Then, a face-to-face interview was done with the parent/caregiver by two of the investigators. A hard copy of the questionnaire was distributed to the participants. Furthermore, the investigators were always available during the completion of the questionnaire, and the participants were encouraged to approach the investigators whenever they needed clarification of any point.

Content of the survey instrument

The survey involved 16 multiple-choice questions written in the patient’s native language, which is Arabic. There were two questions related to participants' demographic information (sex and age group), eight questions regarding their knowledge, four questions about the attitude, and finally two questions regarding perceptions. The survey covered different categories: oral hygiene habits, awareness of gingival health and plaque, knowledge and awareness of dental and general health, and attitudes toward professional dental care. The assessment of oral hygiene habits included brushing frequency and the role of parental supervision.

Ethical consideration

Ethical approval for this study was obtained from the Institutional Review Board (IRB) of King Salman Heart Center, Saudi Arabia (IRB Log No. 22-482).

Sample size

For this cross-sectional study, a total of 112 participants were required based on a response rate of 50%, a confidence interval of 95%, a margin of error of 5%, and a power of 80%. The following formula was used to calculate the sample size: Sample Size = Z 1-α/2 2 SD2 / d2, where Z 1-α/2 is the standard normal variate (representing a 5% type 1 error), SD is the standard deviation of the variable, and d is the absolute error.

Data analysis

Data were analyzed using IBM SPSS Statistics for Windows, Version 26 (Released 2019; IBM Corp., Armonk, New York, United States). Descriptive statistics (frequencies and percentages) was used to describe the categorical variables. To assess the statistical significance of the observed categorical responses regarding eight items of knowledge, four items of attitude, and two items of perception, a nonparametric Pearson's chi-square test for fitness was employed. A p-value of ≤ 0.05 was used to report the statistical significance of the results.

Consistency and validation of the questionnaire

The questionnaire was prepared based on the available information on the World Health Organization (WHO) collaborating center for community oral health programs and research. The questionnaire was reviewed by a panel of experts and revised based on their comments. After that, a pilot study was conducted to evaluate the internal consistency and validity of the Arabic version of the questionnaire by asking 20 participants to answer the translated questionnaire. Cronbach’s alpha was calculated, and it was within the acceptable level (α = 0.74). Most items appeared to be worthy of retention.

## Results

A total of 112 parents responded to this study, which aimed to assess their knowledge and attitudes toward oral hygiene. Out of the 112 parents, 60 (53.6%) provided responses regarding their male child, while the remaining 52 responded for their female child. The distribution of participants based on age was as follows: the smallest group consisted of children aged 0 to 12 months (7.1%), while the most prevalent group consisted of children aged 9 to 12 years (29.5%).

In terms of oral hygiene maintenance, approximately 50% of the parents responded that their children do not brush their teeth, while only 12.5% reported that their children brush their teeth twice a day. Regarding the role of parents in supervising oral hygiene, 62.5% of them stated that they only provide advice but do not watch their child brush their teeth, while 27.7% neither advise nor watch. Only 9.8% of parents are actively involved in advising and watching their children brush their teeth (Table 1).

**Table 1:** Demographics and perception of the study subjects (n=112).

Study variables	No. (%)
Age groups	
0-12 months	8 (7.1)
2-4 years	25 (31.3)
5-8 years	36 (32.1)
9-12 years	33 (29.5)
Gender	
Male	60 (53.6)
Female	52 (46.4)
How many times do you brush your teeth?	
I don’t brush my teeth	56 (50.0)
Once in the morning	9 (8.0)
Once in the night	33 (29.5)
Twice a day in the morning and in the night	14 (12.5)
Role of parents in supervision of oral hygiene	
Watch and advice	11 (9.8)
Only advice but do not watch	70 (62.5)
Parents don’t advice and don’t watch	31 (27.7)

Regarding their knowledge of oral hygiene and awareness of gingival health, 34.8% of parents answered that plaque is soft deposits on teeth that can be removed by manual brushing, which is significantly higher than other two options of responses (p=0.002); 66.1% of them said that gingivitis could be prevented by brushing the teeth and using dental floss, which is significantly higher than other three responses (p<0.0001). Regarding the knowledge of dental and general health, when asked about the effect of sweets on dental health, 97.3% replied in agreement, which is highly statistically significant (p<0.0001), while 2.7% responded with denial. For the awareness of the oral health effect on the cardiac condition, 91.1% were aware of the significance, whereas 8.9% had no clue about the association between them (Table 2).

**Table 2:** Distribution and comparison of parent’s responses related to the items of their knowledge of oral hygiene.

Study variables	No. (%)	Χ^2^ -value	p-value
What does plaque mean?			
Soft deposits on teeth that can be removed by manual brushing	36 (32.1)	15.21	0.002
Heavy deposits on teeth that need to be removed by the dentist	39 (34.8)		
Tooth discoloration	13 (11.6)		
I don’t know	24 (21.4)		
How to prevent gingivitis?			
By brushing the teeth and using dental floss	74 (66.1)	103.07	<0.0001
Taking vitamin C	11 (9.8)		
Eating soft food	8 (7.1)		
I don’t know	19 (17.0)		
Do sweets affect dental health?			
Yes	109 (97.3)	100.32	<0.0001
No	3 (2.7)		
I don’t know	--		
Does discolored teeth affect your child’s appearance?			
Yes	100 (89.3)	69.14	<0.0001
No	12 (10.7)		
I don’t know	--		
Does the health of the mouth and teeth impact the health of body?			
Yes	107 (95.5)	92.89	<0.0001
No	5 (4.5)		
I don’t know	--		
Is treatment of toothache as important as any organ in the body?			
Yes	107 (95.5)	100.32	<0.0001
No	3 (2.7)		
I don’t know	--		
Are regular visits to the dentist necessary?			
Yes	111 (99.1)	108.04	<0.0001
No	1 (0.9)		
I don’t know	--		
Do you know that the condition of a child’s mouth can adversely affect his/her heart?			
Yes	102 (91.1)	75.57	<0.0001
No	--		
I don’t know	10 (8.9)		

On the assessment of the attitude toward professional dental care, 64.3% responded that they visit the dentist only when in pain, which is statistically significantly higher than the other two responses (regularly and occasionally or never) (p<0.0001). Concomitantly, pain happens to be the primary reason for their last visit to the dentist in 75.9% of the respondents. Regarding the reasons for not visiting the dentist, most of them justified it with the high cost (36.6%) among the five other reasons, which is highly statistically significant (p<0.0001). When the parents were asked about their interest in receiving more education about oral health and its impact on overall well-being, 58% of them responded affirmatively, while 22.3% of them were not interested, and 19.7% were neutral (Table 3).

**Table 3:** Distribution and comparison of parent’s responses related to the items of their attitude toward oral hygiene.

Attitudes items	No. (%)	Χ^2^ -value	p-value
How often do you visit the dentist?			
Regularly	13 (11.6)	50.91	<0.0001
When in pain	72 (64.3)		
Occasionally or never	27 (24.1)		
Reasons behind not visiting/dislike visiting the dentist			
Fear	19 (17.0)	20.86	<0.0001
High cost	41 (36.6)		
No clinic nearby	15 (13.4)		
No time	15 (13.4)		
No specific reason	22 (19.6)		
Reason for your last visit to the dentist			
Toothache	85 (75.9)	155.21	<0.0001
Parents advice	6 (5.4)		
Dentists advice	11 (9.8)		
Other reasons	10 (8.9)		
Are you interested in educating yourself about the importance of oral health, its implication on general well-being (especially the heart)?			
Yes	65 (58.0)	30.87	<0.0001
No	25 (22.3)		
I don’t know	22 (19.7)		

## Discussion

It is well established that oral microorganisms play a role in the development of IE [7]. Even under normal physiological conditions, inadequate oral hygiene can lead to bacteremia, increasing the risk of permanent disease acquisition [8]. Previous research has shown that the cardiac group exhibits significantly poorer dental health behaviors compared to the healthy group [12, 15-18]. Therefore, this study aimed to assess the knowledge and preventive measures employed by parents/caregivers of children at risk for IE attending the pediatric outpatient clinics at King Fahad Medical City (KFMC) by distributing a self-constructed questionnaire. Almost half of the participants in this study reported that their children do not brush their teeth regularly. Regarding the role of parents in supervising oral hygiene, more than half of them stated that they only provide advice but do not watch their child brush their teeth. Moreover, almost all of the parents who participated in this study agreed that maintaining good dental health was crucial for overall body health.

This study examined a total of 112 parents where almost half of them provided responses regarding their male child, while the remaining half responded for their female child. The distribution of participants based on their child’s age was as follows: the smallest group consisted of children aged 0 to 12 months (7.1%), while the most prevalent group consisted of children aged 9 to 12 years (29.5%). Roughly, a third of our participants (38.4%) fell within the age range of 0 to 4 years. This aligns with the findings of da Silva et al., who recommended that caregivers of children in this age group receive guidance on performing oral hygiene practices for those who lack the necessary manual dexterity or motivation to maintain proper oral hygiene [17].

In relation to knowledge, almost all of the participants in our study (91.1%) were aware of the relationship between oral health and cardiac condition compared to that reported in the study by da Silva et al., where only 43% of the participants were aware of the relationship. This difference can be attributed to the improvement of comprehensive dental public health programs over the years in Saudi Arabia [19]. Regarding their knowledge of oral hygiene and awareness of gingival health, 66.1% of the participants in this study recognize that gingivitis could be prevented by brushing their teeth and using dental floss. On the other hand, a study done by Al-Omiri et al. found that the majority of their participants failed to recognize the role of tooth brushing in preventing gingivitis [20]. Furthermore, in our study, a substantial majority of our participants were aware of the fact that sweets affect dental health and that health of the oral cavity impacts the health of the body (97.3% and 95.5% respectively). These findings align closely with the results reported by Al-Omiri et al., whose research indicated awareness levels of 87% and 54% among their respective participants [20].

Regarding attitudes and perceptions toward oral hygiene, the participants in this study demonstrated positive attitudes toward their dentists and a high awareness of the link between oral health and systemic well-being, which aligns with the findings of the study by Al-Omiri et al. [20]. However, 75.9% of the respondents in this study stated that they only visit the dentist when their child is in pain, which may be attributed to the high cost of treatment. This finding contrasts with the study by Al-Omiri et al., where fear was reported as the topmost reason for not visiting the dentist [20]. Almost 50% of the participants in this study reported that their children do not brush their teeth regularly, with only 12.5% of them brushing twice a day. This can be attributed to the lack of parental encouragement as most parents in this study reported that they only advise their children but do not supervise their tooth brushing. This parental negligence of oral hygiene may be influenced by a greater concern for their child's cardiac condition, as Hallett et al. and da Silva et al. stated in their studies [12,17]. On the other hand, Suvarna et al. reported a high percentage of children who are more conscientious about brushing their teeth [16]. It is well established in multiple studies that children with CHD are strongly advised to receive thorough dental care, especially at a young age [15]. The findings are rather surprising in the sense that a significant number of participants demonstrated ample awareness of the importance of regular visits to the dentist, as mentioned in multiple studies [15-18]. The majority of people were aware that poor oral health poses risks to their cardiovascular health. However, despite this knowledge, they did not adhere to the recommendations in the literature for regular dental visits. Due to the growing count of children who are surviving IE, it becomes crucial to formulate dental care that caters to their specific requirements. The findings suggest that it is advisable for these children to undergo dental treatment at pediatric dentistry clinics, especially during their early years.

Limitations

Limitations of the current study were the lack of oral examination to evaluate the oral health status or the quality of tooth brushing. Additionally, the study was conducted for a short duration, and the sample size was relatively small.

## Conclusions

Parents in our study recognized the importance of dental health but made limited efforts to translate this understanding into practice. Therefore, there is a need to improve the attitudes of parents and children toward oral health and dental care by implementing comprehensive educational programs that specifically address oral health for both parents and their children. Fortunately, the majority of participants expressed willingness to receive further education on oral health.
